# Construction of a dinuclear silver(I) coordination complex with a Schiff base containing 4-amino-1,2,4-triazole ligands

**DOI:** 10.1107/S1600536809004760

**Published:** 2009-02-18

**Authors:** Qiaozhen Sun, Feng Zheng, Xiaodan Sun, Wei Wang

**Affiliations:** a Department of Materials Chemistry, School of Materials Science and Engineering, Key Laboratory of Non-ferrous Metals of the Ministry of Education, Central South University, Changsha 410083, People’s Republic of China

## Abstract

The new ligand 1-(1,2,4-triazol-4-ylimino­meth­yl)-2-naphthol (*L*) and the title silver(I) complex, namely bis­[μ-1-(1,2,4-triazol-4-ylimino­meth­yl)-2-naphthol]bis­{[1-(1,2,4-triazol-4-yl­imino­meth­yl)-2-naphthol]silver(I)} dinitrate monohydrate, [Ag_2_(C_13_H_10_N_4_O)_4_](NO_3_)_2_·H_2_O, were synthesized. Each silver center in the dimeric complex (related by an inversion centre) is coordinated by two bridging *L* ligands and one additional *L* ligand in a monodentate fashion, exhibiting a distorted trigonal-planar coordination. The discrete dimers are further linked through O—H⋯O hydrogen bonds and weak π–π stacking inter­actions [the shortest atom–atom separation is *ca* 3.46 Å between the parallel stacking pairs]. Intramolecular O—H⋯N hydrogen bonds are also present.

## Related literature

For the structures of other triazole Schiff base compounds, see: Beckmann & Brooker (2003 ▶); Drabent *et al.* (2003 ▶, 2004 ▶); Garcia *et al.* (1997 ▶); Klingele & Brooker (2003 ▶); Liu *et al.* (2003 ▶, 2006 ▶); Wang *et al.* (2006 ▶); Yi *et al.* (2004 ▶); Zhai *et al.* (2006 ▶). For related literature, see: Han *et al.* (2004 ▶).
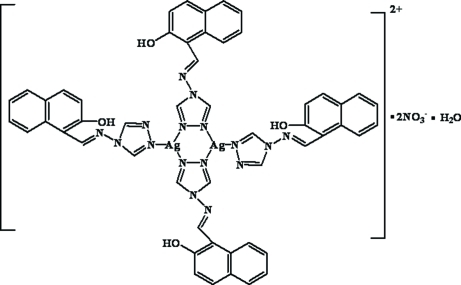


## Experimental

### 

#### Crystal data

[Ag_2_(C_13_H_10_N_4_O)_4_](NO_3_)_2_·H_2_O
*M* *_r_* = 1310.78
Triclinic, 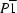

*a* = 9.8594 (15) Å
*b* = 10.7081 (15) Å
*c* = 12.8567 (19) Å
α = 82.391 (2)°
β = 81.155 (2)°
γ = 77.626 (2)°
*V* = 1303.1 (3) Å^3^
*Z* = 1
Mo *K*α radiation
μ = 0.83 mm^−1^
*T* = 293 K
0.20 × 0.18 × 0.16 mm

#### Data collection

Bruker APEX CCD area-detector diffractometer
Absorption correction: multi-scan (*SADABS*; Bruker, 2000 ▶) *T*_min_ = 0.826, *T*_max_ = 0.887
6610 measured reflections
4536 independent reflections
3137 reflections with *I* > 2σ(*I*)
*R*_int_ = 0.118

#### Refinement

*R*[*F* ^2^ > 2σ(*F* ^2^)] = 0.055
*wR*(*F* ^2^) = 0.133
*S* = 1.00
4536 reflections
379 parameters
H-atom parameters constrained
Δρ_max_ = 0.85 e Å^−3^
Δρ_min_ = −0.84 e Å^−3^

### 

Data collection: *SMART* (Bruker, 2000 ▶); cell refinement: *SAINT* (Bruker, 2000 ▶); data reduction: *SAINT*; program(s) used to solve structure: *SHELXTL* (Sheldrick, 2008 ▶); program(s) used to refine structure: *SHELXTL*; molecular graphics: *SHELXTL*; software used to prepare material for publication: *SHELXTL*.

## Supplementary Material

Crystal structure: contains datablocks global, I. DOI: 10.1107/S1600536809004760/wk2097sup1.cif

Structure factors: contains datablocks I. DOI: 10.1107/S1600536809004760/wk2097Isup2.hkl

Additional supplementary materials:  crystallographic information; 3D view; checkCIF report

## Figures and Tables

**Table 1:** Hydrogen-bond geometry (Å, °)

*D*—H⋯*A*	*D*—H	H⋯*A*	*D*⋯*A*	*D*—H⋯*A*
O1—H1*A*⋯N1	0.82	1.83	2.548 (4)	145
O2—H2*B*⋯N5	0.82	1.87	2.588 (5)	145
O1*W*—H1*WA*⋯O3^i^	0.85	1.85	2.594 (15)	145

Symmetry code: (i) 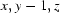.

**Fig. 1.**
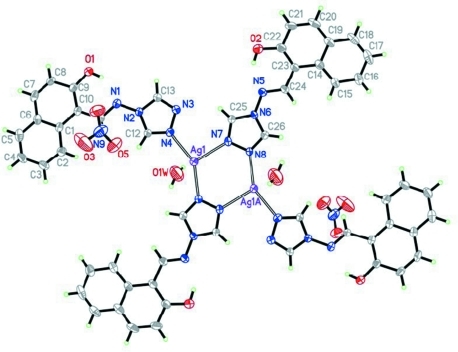
A perspective view of the molecular structure showing the atom-numbering scheme. Displacement ellipsoids are drawn at the 30% probability level. [symmetry code: (A) 2 - x, -y, 2 - z.]

**Fig. 2.**
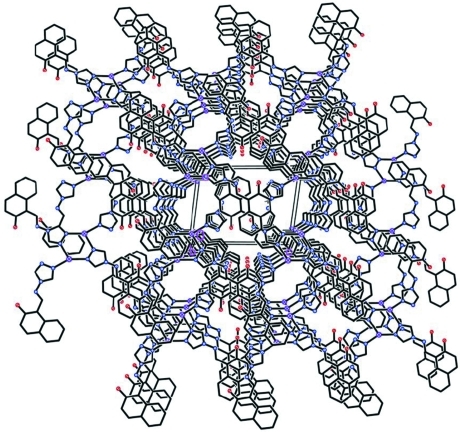
A packing diagram for the crystal along b axis, the NO3-, H2O and hydrogen atoms are omitted for clarity.

## supplementary crystallographic information

### Comment

1,2,4-triazoles and their derivatives are interesting bridging ligands. 1,2,4-triazoles can coordinate with metals by bridging two adjacent nitrogen atoms (N1 and N2) or via the 4-positioned one (N4). It is also a readily available and inexpensive resource. In the past two decades, a variety of coordination compounds containing 1,2,4-triazoles, N4 substituted 1,2,4-triazoles and their derivatives as ligands coordinated to metal ions have been reported (Beckmann & Brooker, 2003; Garcia *et al.*, 1997; Klingele & Brooker, 2003; Liu *et al.*, 2006; Liu *et al.*, 2003; Yi *et al.*, 2004; Zhai *et al.*, 2006). Relatively few structurally characterized compounds based on 4-amido-1,2,4-triazoles Schiff base ligands have been reported (Drabent *et al.*, 2004 and 2003; Wang *et al.*, 2006). Here we describe the synthesis of the Ag(I) metal complex with a Schiff-base containing triazole ligand.

The molecular structure of complex 1 is shown in Figure 1. It consists of a discrete binuclear complex of Ag(I) bridged by two N1,N2-coordinated triazole ligands and an additional triazole ligand is bound to the Ag(I) ion in a monodentate fashion. This coordination mode results in a trigonal planar coordination environment (the sum of the angles around Ag metal atom is equal to 360 °). The  Ag—Ag distance is equal to 3.81 Å, which is over the summed van der Waals radii of two Ag(I) atoms (3.44 Å) (Han *et al.*, 2004). The Ag—N bond distances are in the range of 2.18–2.33 Å. The six-membered Ag-[N—N]_2_-Ag rings remain almost planar (the mean plane deviation is 0.06 Å) from planarity, which is similar withdinuclear Cu(I) complex (Drabent *et al.*, 2004). The Ag—N—N—Ag dihedral angle is 15.1 °.

In this complex all the ligands *L* are coordinated in almost planar E configuration and the resulting binuclear units can be described as X-shaped dimers. Sheets formed through C-H···O hydrogen bonds are further aggregated into three-dimensions by weak π-π stacking interactions between the naphthyl rings of the neighbouring dimers in different sheets and the shortest atom···atom separation is *ca* 3.46 Å between the parallel stacking pairs. The anions and water molecules interact with one another through O—H···N, O—H···O hydrogen bonds.

### Experimental

Preparation of complex 1: The ligand *L* (0.1 mmol, 0.024 g) and AgNO_3_ (0.1 mmol, 0.017 g) were mixed in acetonitrile and stirred at room temperature for one hour, the yellow solution was filtered and evaporated at room temperature. A few days later orange block crystals were obtained.

Preparation of the ligand *L*: An ethanolic solution (20 ml) of 2-hydroxy-1-napthaldehyde (1.72 g, 10 mmol) was added to a warm ethanolic solution (10 ml) of 4-amino- 1,2,4-triazole (0.84 g, 10 mmol) and the resulting solution was refluxed for four hours. The reaction mixture was then cooled to room temperature. Upon standing overnight the resultant yellow solid was filtered off, washed with diethyl ether and dried under vacuum. Yield: 90%. ^1^H NMR (500 MHz, DMSO, 298 K): 9.66 (s, 1H), 9.34 (s, 2H), 8.84–8.86 (d, 1H), 8.05–8.07 (d, 1H), 7.90–7.92 (d, 1H), 7.61–7.64 (t, 1H), 7.43–7.46 (t, 1H), 7.28–7.30 (d, 1H).

### Refinement

All of the non-hydrogen atoms were refined with anisotropic thermal displacement coefficients. The positions of hydrogen atoms were fixed geometrically at calculated distances and allowed to ride on the parent non-hydrogen atoms. The water molecule was refined as disordered with the s.o.f. being fixed at 0.5 and its hydrogen atoms located in the difference Fourier maps and fixed at calculated distances from the parent oxygen atom.

### Crystal data

**Table:**

[Ag_2_(C_13_H_10_N_4_O)_4_](NO_3_)_2_·H_2_O	*Z* = 1
*M**_r_* = 1310.78	*F*(000) = 662
Triclinic, *P*1	*D*_x_ = 1.670 Mg m^−^^3^
Hall symbol: -P 1	Mo *K*α radiation, λ = 0.71073 Å
*a* = 9.8594 (15) Å	Cell parameters from 2250 reflections
*b* = 10.7081 (15) Å	θ = 2.4–25.4°
*c* = 12.8567 (19) Å	µ = 0.83 mm^−^^1^
α = 82.391 (2)°	*T* = 293 K
β = 81.155 (2)°	Block, orange
γ = 77.626 (2)°	0.2 × 0.18 × 0.16 mm
*V* = 1303.1 (3) Å^3^	

### Data collection

**Table:**

Bruker APEX CCD area-detector diffractometer	4536 independent reflections
Radiation source: fine-focus sealed tube	3137 reflections with *I* > 2σ(*I*)
graphite	*R*_int_ = 0.118
φ and ω scans	θ_max_ = 25.0°, θ_min_ = 1.6°
Absorption correction: multi-scan (*SADABS*; Bruker, 2000)	*h* = −10→11
*T*_min_ = 0.826, *T*_max_ = 0.887	*k* = −12→8
6610 measured reflections	*l* = −15→14

### Refinement

**Table:**

Refinement on *F*^2^	Primary atom site location: structure-invariant direct methods
Least-squares matrix: full	Secondary atom site location: difference Fourier map
*R*[*F*^2^ > 2σ(*F*^2^)] = 0.055	Hydrogen site location: geom, H2O from difmap
*wR*(*F*^2^) = 0.133	H-atom parameters constrained
*S* = 1.00	*w* = 1/[σ^2^(*F*_o_^2^) + (0.0601*P*)^2^] where *P* = (*F*_o_^2^ + 2*F*_c_^2^)/3
4536 reflections	(Δ/σ)_max_ = 0.001
379 parameters	Δρ_max_ = 0.85 e Å^−^^3^
0 restraints	Δρ_min_ = −0.84 e Å^−^^3^

### Special details

**Table:**

Geometry. All e.s.d.'s (except the e.s.d. in the dihedral angle between two l.s. planes) are estimated using the full covariance matrix. The cell e.s.d.'s are taken into account individually in the estimation of e.s.d.'s in distances, angles and torsion angles; correlations between e.s.d.'s in cell parameters are only used when they are defined by crystal symmetry. An approximate (isotropic) treatment of cell e.s.d.'s is used for estimating e.s.d.'s involving l.s. planes.
Refinement. Refinement of *F*^2^ against ALL reflections. The weighted *R*-factor *wR* and goodness of fit *S* are based on *F*^2^, conventional *R*-factors *R* are based on *F*, with *F* set to zero for negative *F*^2^. The threshold expression of *F*^2^ > σ(*F*^2^) is used only for calculating *R*-factors(gt) *etc*. and is not relevant to the choice of reflections for refinement. *R*-factors based on *F*^2^ are statistically about twice as large as those based on *F*, and *R*- factors based on ALL data will be even larger.

### Fractional atomic coordinates and isotropic or equivalent isotropic displacement parameters (Å^2^)

**Table:**

	*x*	*y*	*z*	*U*_iso_*/*U*_eq_	Occ. (<1)
Ag1	0.84563 (4)	0.12190 (4)	0.95202 (3)	0.0613 (2)	
O1	0.3147 (4)	0.5935 (3)	0.5781 (2)	0.0564 (9)	
H1A	0.3862	0.5514	0.6002	0.085*	
O2	1.2327 (4)	0.0054 (4)	0.4026 (3)	0.0701 (11)	
H2B	1.2018	0.0029	0.4656	0.105*	
N1	0.4657 (4)	0.4732 (4)	0.7179 (3)	0.0488 (10)	
N2	0.5888 (4)	0.3908 (4)	0.7472 (3)	0.0447 (10)	
N3	0.7966 (4)	0.2720 (4)	0.7236 (3)	0.0590 (12)	
N4	0.7427 (4)	0.2564 (4)	0.8288 (3)	0.0554 (11)	
N5	1.2436 (4)	−0.0497 (4)	0.6037 (3)	0.0453 (9)	
N6	1.1907 (4)	−0.0406 (3)	0.7095 (3)	0.0412 (9)	
N7	1.0373 (4)	0.0221 (4)	0.8420 (3)	0.0489 (10)	
N8	1.1554 (4)	−0.0524 (4)	0.8806 (3)	0.0447 (10)	
C1	0.1290 (5)	0.6319 (4)	0.8481 (4)	0.0423 (11)	
C2	0.1404 (6)	0.6048 (5)	0.9573 (4)	0.0530 (13)	
H2A	0.2232	0.5561	0.9789	0.064*	
C3	0.0338 (6)	0.6481 (6)	1.0306 (4)	0.0633 (15)	
H3A	0.0449	0.6287	1.1019	0.076*	
C4	−0.0937 (6)	0.7218 (6)	1.0028 (4)	0.0663 (15)	
H4A	−0.1664	0.7506	1.0545	0.080*	
C5	−0.1082 (6)	0.7501 (5)	0.8987 (5)	0.0650 (15)	
H5A	−0.1922	0.7993	0.8794	0.078*	
C6	−0.0002 (5)	0.7072 (5)	0.8189 (4)	0.0514 (12)	
C7	−0.0158 (6)	0.7374 (5)	0.7109 (4)	0.0582 (14)	
H7A	−0.1003	0.7859	0.6921	0.070*	
C8	0.0863 (6)	0.6988 (5)	0.6352 (4)	0.0552 (14)	
H8A	0.0722	0.7200	0.5646	0.066*	
C9	0.2163 (5)	0.6255 (4)	0.6608 (4)	0.0442 (11)	
C10	0.2382 (5)	0.5910 (4)	0.7651 (4)	0.0404 (11)	
C11	0.3702 (5)	0.5102 (4)	0.7907 (4)	0.0419 (11)	
H11A	0.3840	0.4858	0.8611	0.050*	
C12	0.6178 (5)	0.3277 (5)	0.8406 (4)	0.0515 (13)	
H12A	0.5582	0.3338	0.9040	0.062*	
C13	0.7004 (5)	0.3530 (5)	0.6776 (4)	0.0548 (14)	
H13A	0.7082	0.3809	0.6057	0.066*	
C14	1.5717 (5)	−0.2019 (5)	0.4532 (4)	0.0459 (11)	
C15	1.6506 (5)	−0.2714 (5)	0.5336 (4)	0.0570 (13)	
H15A	1.6110	−0.2711	0.6041	0.068*	
C16	1.7841 (6)	−0.3386 (6)	0.5089 (5)	0.0684 (16)	
H16A	1.8327	−0.3846	0.5629	0.082*	
C17	1.8483 (7)	−0.3395 (6)	0.4050 (6)	0.083 (2)	
H17A	1.9405	−0.3821	0.3897	0.099*	
C18	1.7744 (7)	−0.2771 (6)	0.3256 (5)	0.0719 (17)	
H18A	1.8153	−0.2810	0.2556	0.086*	
C19	1.6372 (6)	−0.2068 (5)	0.3479 (4)	0.0577 (14)	
C20	1.5622 (7)	−0.1409 (6)	0.2656 (4)	0.0679 (17)	
H20A	1.6040	−0.1457	0.1959	0.082*	
C21	1.4303 (7)	−0.0703 (6)	0.2851 (4)	0.0673 (17)	
H21A	1.3840	−0.0259	0.2294	0.081*	
C22	1.3641 (6)	−0.0650 (5)	0.3907 (4)	0.0526 (13)	
C23	1.4329 (5)	−0.1275 (4)	0.4742 (3)	0.0435 (11)	
C24	1.3675 (5)	−0.1161 (5)	0.5824 (3)	0.0441 (11)	
H24A	1.4163	−0.1578	0.6377	0.053*	
C25	1.0619 (5)	0.0279 (5)	0.7388 (4)	0.0477 (12)	
H25A	1.0000	0.0725	0.6926	0.057*	
C26	1.2458 (5)	−0.0883 (4)	0.7999 (3)	0.0432 (11)	
H26A	1.3346	−0.1390	0.8039	0.052*	
N9	0.5609 (6)	0.7008 (7)	0.8823 (5)	0.0847 (17)	
O3	0.5165 (10)	0.7921 (8)	0.9419 (6)	0.183 (4)	
O4	0.5557 (6)	0.7361 (6)	0.7950 (4)	0.125 (2)	
O5	0.5869 (6)	0.5986 (5)	0.9295 (5)	0.116 (2)	
O1W	0.6044 (13)	−0.0162 (13)	0.9945 (10)	0.143 (5)	0.50
H1WA	0.6068	−0.0783	0.9589	0.171*	0.50
H1WB	0.5600	−0.0207	1.0566	0.171*	0.50

### Atomic displacement parameters (Å^2^)

**Table:**

	*U*^11^	*U*^22^	*U*^33^	*U*^12^	*U*^13^	*U*^23^
Ag1	0.0486 (3)	0.0810 (4)	0.0381 (2)	0.0158 (2)	−0.00257 (16)	0.00200 (18)
O1	0.053 (2)	0.067 (2)	0.0399 (17)	0.0063 (18)	−0.0092 (16)	0.0002 (16)
O2	0.076 (3)	0.077 (3)	0.0453 (19)	0.013 (2)	−0.0091 (18)	−0.0055 (18)
N1	0.041 (2)	0.054 (3)	0.046 (2)	0.005 (2)	−0.0109 (19)	−0.0009 (19)
N2	0.037 (2)	0.051 (2)	0.039 (2)	0.0072 (18)	−0.0074 (17)	−0.0035 (17)
N3	0.046 (3)	0.076 (3)	0.041 (2)	0.011 (2)	−0.0012 (19)	−0.001 (2)
N4	0.048 (3)	0.073 (3)	0.037 (2)	0.009 (2)	−0.0116 (19)	−0.001 (2)
N5	0.047 (2)	0.046 (2)	0.0352 (19)	0.0018 (19)	0.0022 (17)	−0.0045 (16)
N6	0.038 (2)	0.041 (2)	0.0361 (19)	0.0033 (18)	0.0052 (16)	−0.0051 (16)
N7	0.038 (2)	0.054 (3)	0.043 (2)	0.0105 (19)	−0.0008 (17)	−0.0038 (18)
N8	0.037 (2)	0.053 (3)	0.036 (2)	0.0062 (18)	−0.0033 (17)	−0.0029 (17)
C1	0.036 (3)	0.039 (3)	0.051 (3)	−0.003 (2)	−0.010 (2)	−0.004 (2)
C2	0.045 (3)	0.061 (3)	0.052 (3)	−0.008 (3)	−0.011 (2)	−0.003 (2)
C3	0.058 (4)	0.087 (4)	0.044 (3)	−0.017 (3)	0.006 (2)	−0.013 (3)
C4	0.049 (3)	0.082 (4)	0.063 (3)	−0.004 (3)	0.009 (3)	−0.022 (3)
C5	0.041 (3)	0.060 (4)	0.089 (4)	0.001 (3)	−0.005 (3)	−0.012 (3)
C6	0.035 (3)	0.051 (3)	0.066 (3)	−0.002 (2)	−0.009 (2)	−0.008 (2)
C7	0.043 (3)	0.058 (3)	0.070 (4)	0.005 (3)	−0.024 (3)	−0.001 (3)
C8	0.056 (3)	0.056 (3)	0.049 (3)	0.006 (3)	−0.022 (3)	0.000 (2)
C9	0.046 (3)	0.041 (3)	0.045 (3)	−0.003 (2)	−0.011 (2)	−0.001 (2)
C10	0.034 (3)	0.036 (3)	0.051 (3)	−0.003 (2)	−0.011 (2)	−0.005 (2)
C11	0.037 (3)	0.046 (3)	0.039 (2)	0.000 (2)	−0.007 (2)	−0.005 (2)
C12	0.042 (3)	0.067 (3)	0.035 (2)	0.014 (2)	−0.008 (2)	−0.006 (2)
C13	0.045 (3)	0.071 (4)	0.036 (2)	0.009 (3)	−0.001 (2)	0.002 (2)
C14	0.048 (3)	0.043 (3)	0.048 (3)	−0.014 (2)	0.005 (2)	−0.014 (2)
C15	0.048 (3)	0.058 (3)	0.062 (3)	−0.004 (3)	0.006 (3)	−0.019 (3)
C16	0.041 (3)	0.076 (4)	0.086 (4)	−0.004 (3)	−0.001 (3)	−0.022 (3)
C17	0.053 (4)	0.079 (5)	0.109 (5)	−0.007 (3)	0.025 (4)	−0.040 (4)
C18	0.068 (4)	0.076 (4)	0.069 (4)	−0.024 (3)	0.031 (3)	−0.029 (3)
C19	0.062 (4)	0.056 (3)	0.055 (3)	−0.022 (3)	0.018 (3)	−0.020 (3)
C20	0.085 (5)	0.074 (4)	0.042 (3)	−0.027 (4)	0.022 (3)	−0.014 (3)
C21	0.097 (5)	0.068 (4)	0.033 (3)	−0.014 (4)	−0.002 (3)	−0.002 (2)
C22	0.063 (4)	0.048 (3)	0.043 (3)	−0.008 (3)	0.003 (2)	−0.008 (2)
C23	0.049 (3)	0.040 (3)	0.039 (2)	−0.010 (2)	0.005 (2)	−0.008 (2)
C24	0.042 (3)	0.048 (3)	0.039 (2)	−0.006 (2)	0.003 (2)	−0.003 (2)
C25	0.038 (3)	0.055 (3)	0.039 (2)	0.007 (2)	−0.002 (2)	0.001 (2)
C26	0.041 (3)	0.047 (3)	0.036 (2)	0.002 (2)	−0.005 (2)	−0.004 (2)
N9	0.076 (4)	0.099 (5)	0.081 (4)	−0.028 (4)	−0.029 (3)	0.021 (4)
O3	0.216 (9)	0.163 (7)	0.149 (6)	−0.041 (6)	0.080 (6)	−0.057 (6)
O4	0.101 (4)	0.177 (6)	0.076 (3)	−0.002 (4)	−0.015 (3)	0.031 (4)
O5	0.132 (5)	0.090 (4)	0.118 (4)	−0.007 (3)	−0.041 (4)	0.027 (3)
O1W	0.115 (10)	0.181 (12)	0.125 (9)	−0.003 (9)	0.005 (8)	−0.057 (8)

### Geometric parameters (Å, °)

**Table:**

Ag1—N8^i^	2.182 (3)	C7—H7A	0.9300
Ag1—N4	2.209 (4)	C8—C9	1.413 (6)
Ag1—N7	2.329 (4)	C8—H8A	0.9300
O1—C9	1.351 (6)	C9—C10	1.381 (6)
O1—H1A	0.8200	C10—C11	1.458 (6)
O2—C22	1.349 (7)	C11—H11A	0.9300
O2—H2B	0.8200	C12—H12A	0.9300
N1—C11	1.259 (6)	C13—H13A	0.9300
N1—N2	1.410 (5)	C14—C19	1.410 (6)
N2—C13	1.333 (6)	C14—C15	1.422 (7)
N2—C12	1.338 (5)	C14—C23	1.434 (7)
N3—C13	1.298 (6)	C15—C16	1.370 (8)
N3—N4	1.377 (5)	C15—H15A	0.9300
N4—C12	1.300 (6)	C16—C17	1.388 (9)
N5—C24	1.284 (6)	C16—H16A	0.9300
N5—N6	1.389 (5)	C17—C18	1.363 (9)
N6—C25	1.349 (6)	C17—H17A	0.9300
N6—C26	1.351 (5)	C18—C19	1.407 (8)
N7—C25	1.307 (6)	C18—H18A	0.9300
N7—N8	1.383 (5)	C19—C20	1.409 (8)
N8—C26	1.301 (6)	C20—C21	1.362 (9)
N8—Ag1^i^	2.182 (3)	C20—H20A	0.9300
C1—C2	1.412 (6)	C21—C22	1.416 (7)
C1—C6	1.428 (6)	C21—H21A	0.9300
C1—C10	1.435 (6)	C22—C23	1.378 (7)
C2—C3	1.346 (8)	C23—C24	1.450 (6)
C2—H2A	0.9300	C24—H24A	0.9300
C3—C4	1.403 (8)	C25—H25A	0.9300
C3—H3A	0.9300	C26—H26A	0.9300
C4—C5	1.355 (8)	N9—O4	1.141 (6)
C4—H4A	0.9300	N9—O5	1.176 (7)
C5—C6	1.406 (7)	N9—O3	1.284 (9)
C5—H5A	0.9300	O1W—H1WA	0.8499
C6—C7	1.408 (7)	O1W—H1WB	0.8500
C7—C8	1.326 (8)		
			
N8^i^—Ag1—N4	147.87 (16)	N1—C11—C10	120.2 (4)
N8^i^—Ag1—N7	114.48 (13)	N1—C11—H11A	119.9
N4—Ag1—N7	97.64 (14)	C10—C11—H11A	119.9
C9—O1—H1A	109.5	N4—C12—N2	109.3 (4)
C22—O2—H2B	109.5	N4—C12—H12A	125.4
C11—N1—N2	117.8 (4)	N2—C12—H12A	125.4
C13—N2—C12	106.0 (4)	N3—C13—N2	111.0 (4)
C13—N2—N1	123.0 (4)	N3—C13—H13A	124.5
C12—N2—N1	130.8 (4)	N2—C13—H13A	124.5
C13—N3—N4	105.8 (4)	C19—C14—C15	116.7 (5)
C12—N4—N3	108.0 (4)	C19—C14—C23	119.6 (5)
C12—N4—Ag1	126.5 (3)	C15—C14—C23	123.7 (4)
N3—N4—Ag1	125.4 (3)	C16—C15—C14	121.1 (5)
C24—N5—N6	117.6 (4)	C16—C15—H15A	119.5
C25—N6—C26	106.3 (4)	C14—C15—H15A	119.5
C25—N6—N5	121.5 (4)	C15—C16—C17	121.4 (6)
C26—N6—N5	132.2 (4)	C15—C16—H16A	119.3
C25—N7—N8	106.9 (4)	C17—C16—H16A	119.3
C25—N7—Ag1	130.5 (3)	C18—C17—C16	119.1 (6)
N8—N7—Ag1	122.5 (3)	C18—C17—H17A	120.4
C26—N8—N7	107.8 (3)	C16—C17—H17A	120.4
C26—N8—Ag1^i^	129.5 (3)	C17—C18—C19	121.0 (6)
N7—N8—Ag1^i^	121.5 (3)	C17—C18—H18A	119.5
C2—C1—C6	117.2 (4)	C19—C18—H18A	119.5
C2—C1—C10	124.7 (4)	C18—C19—C20	120.7 (5)
C6—C1—C10	118.1 (4)	C18—C19—C14	120.6 (6)
C3—C2—C1	121.2 (5)	C20—C19—C14	118.7 (5)
C3—C2—H2A	119.4	C21—C20—C19	121.9 (5)
C1—C2—H2A	119.4	C21—C20—H20A	119.1
C2—C3—C4	121.9 (5)	C19—C20—H20A	119.1
C2—C3—H3A	119.0	C20—C21—C22	119.6 (5)
C4—C3—H3A	119.0	C20—C21—H21A	120.2
C5—C4—C3	118.5 (5)	C22—C21—H21A	120.2
C5—C4—H4A	120.8	O2—C22—C23	123.6 (5)
C3—C4—H4A	120.8	O2—C22—C21	115.6 (5)
C4—C5—C6	121.9 (5)	C23—C22—C21	120.8 (5)
C4—C5—H5A	119.0	C22—C23—C14	119.4 (4)
C6—C5—H5A	119.0	C22—C23—C24	120.6 (5)
C5—C6—C7	121.6 (5)	C14—C23—C24	120.0 (4)
C5—C6—C1	119.2 (5)	N5—C24—C23	121.5 (4)
C7—C6—C1	119.2 (5)	N5—C24—H24A	119.3
C8—C7—C6	121.9 (5)	C23—C24—H24A	119.3
C8—C7—H7A	119.0	N7—C25—N6	109.7 (4)
C6—C7—H7A	119.0	N7—C25—H25A	125.2
C7—C8—C9	120.6 (4)	N6—C25—H25A	125.2
C7—C8—H8A	119.7	N8—C26—N6	109.3 (4)
C9—C8—H8A	119.7	N8—C26—H26A	125.3
O1—C9—C10	123.3 (4)	N6—C26—H26A	125.3
O1—C9—C8	116.2 (4)	O4—N9—O5	134.0 (9)
C10—C9—C8	120.5 (5)	O4—N9—O3	112.1 (8)
C9—C10—C1	119.7 (4)	O5—N9—O3	113.6 (7)
C9—C10—C11	120.1 (4)	H1WA—O1W—H1WB	115.9
C1—C10—C11	120.2 (4)		
			
C11—N1—N2—C13	−175.1 (5)	C1—C10—C11—N1	179.3 (4)
C11—N1—N2—C12	10.7 (8)	N3—N4—C12—N2	−0.9 (6)
C13—N3—N4—C12	0.2 (6)	Ag1—N4—C12—N2	−176.5 (3)
C13—N3—N4—Ag1	175.9 (4)	C13—N2—C12—N4	1.1 (6)
N8^i^—Ag1—N4—C12	−11.8 (6)	N1—N2—C12—N4	176.1 (5)
N7—Ag1—N4—C12	170.0 (5)	N4—N3—C13—N2	0.5 (6)
N8^i^—Ag1—N4—N3	173.4 (3)	C12—N2—C13—N3	−1.0 (6)
N7—Ag1—N4—N3	−4.9 (4)	N1—N2—C13—N3	−176.5 (4)
C24—N5—N6—C25	179.6 (4)	C19—C14—C15—C16	0.5 (8)
C24—N5—N6—C26	1.0 (7)	C23—C14—C15—C16	−178.6 (5)
N8^i^—Ag1—N7—C25	170.2 (4)	C14—C15—C16—C17	1.3 (9)
N4—Ag1—N7—C25	−10.8 (5)	C15—C16—C17—C18	−3.2 (10)
N8^i^—Ag1—N7—N8	−14.1 (4)	C16—C17—C18—C19	3.3 (9)
N4—Ag1—N7—N8	164.8 (3)	C17—C18—C19—C20	179.0 (6)
C25—N7—N8—C26	0.0 (5)	C17—C18—C19—C14	−1.5 (9)
Ag1—N7—N8—C26	−176.6 (3)	C15—C14—C19—C18	−0.3 (7)
C25—N7—N8—Ag1^i^	−168.3 (3)	C23—C14—C19—C18	178.7 (5)
Ag1—N7—N8—Ag1^i^	15.1 (5)	C15—C14—C19—C20	179.1 (5)
C6—C1—C2—C3	0.0 (7)	C23—C14—C19—C20	−1.8 (7)
C10—C1—C2—C3	179.0 (5)	C18—C19—C20—C21	−178.8 (6)
C1—C2—C3—C4	0.1 (9)	C14—C19—C20—C21	1.7 (9)
C2—C3—C4—C5	−0.4 (9)	C19—C20—C21—C22	−1.7 (9)
C3—C4—C5—C6	0.4 (9)	C20—C21—C22—O2	−178.9 (5)
C4—C5—C6—C7	−179.7 (5)	C20—C21—C22—C23	1.8 (9)
C4—C5—C6—C1	−0.2 (8)	O2—C22—C23—C14	178.8 (5)
C2—C1—C6—C5	0.0 (7)	C21—C22—C23—C14	−1.9 (8)
C10—C1—C6—C5	−179.1 (5)	O2—C22—C23—C24	−2.5 (8)
C2—C1—C6—C7	179.5 (5)	C21—C22—C23—C24	176.7 (5)
C10—C1—C6—C7	0.5 (7)	C19—C14—C23—C22	2.0 (7)
C5—C6—C7—C8	179.1 (5)	C15—C14—C23—C22	−179.0 (5)
C1—C6—C7—C8	−0.5 (8)	C19—C14—C23—C24	−176.7 (4)
C6—C7—C8—C9	−0.3 (9)	C15—C14—C23—C24	2.3 (7)
C7—C8—C9—O1	−178.4 (5)	N6—N5—C24—C23	−178.0 (4)
C7—C8—C9—C10	1.2 (8)	C22—C23—C24—N5	1.1 (7)
O1—C9—C10—C1	178.4 (4)	C14—C23—C24—N5	179.7 (5)
C8—C9—C10—C1	−1.2 (7)	N8—N7—C25—N6	0.4 (6)
O1—C9—C10—C11	−3.4 (7)	Ag1—N7—C25—N6	176.6 (3)
C8—C9—C10—C11	177.0 (4)	C26—N6—C25—N7	−0.7 (6)
C2—C1—C10—C9	−178.6 (5)	N5—N6—C25—N7	−179.5 (4)
C6—C1—C10—C9	0.3 (7)	N7—N8—C26—N6	−0.4 (5)
C2—C1—C10—C11	3.2 (7)	Ag1^i^—N8—C26—N6	166.7 (3)
C6—C1—C10—C11	−177.8 (4)	C25—N6—C26—N8	0.7 (6)
N2—N1—C11—C10	−177.4 (4)	N5—N6—C26—N8	179.4 (4)
C9—C10—C11—N1	1.2 (7)		

Symmetry codes: (i) −*x*+2, −*y*, −*z*+2.

### Hydrogen-bond geometry (Å, °)

**Table:**

*D*—H···*A*	*D*—H	H···*A*	*D*···*A*	*D*—H···*A*
O1—H1A···N1	0.82	1.83	2.548 (4)	145
O2—H2B···N5	0.82	1.87	2.588 (5)	145
O1W—H1WA···O3^ii^	0.85	1.85	2.594 (15)	145

Symmetry codes: (ii) *x*, *y*−1, *z*.
